# Uterine perforation following a fractional curettage successfully treated with the modified polysaccharide 4DryField® PH: a case report

**DOI:** 10.1186/s13256-016-1029-x

**Published:** 2016-09-06

**Authors:** Nicole Ziegler, Matthias Korell, Anja Herrmann, Maya Sophie de Wilde, Luz Angela Torres-de la Roche, Angelika Larbig, Rudy Leon De Wilde

**Affiliations:** 1 Clinic of Gynecology, Obstetrics and Gynecological Oncology, University Hospital for Gynecology, Pius Hospital-Medical Campus University, Oldenburg, Germany; 2 Department of Obstetrics and Gynecology, Johanna-Etienne-Hospital, Neuss, Germany

**Keywords:** Uterine perforation, Hemostatics, Adhesions, Polysaccharide, Case report

## Abstract

**Background:**

Uterine perforation is the most common complication of curettage and may result in bleeding. Therefore, urgent control of bleeding from the uterine wall perforation is necessary to avoid an emergency hysterectomy or blood transfusion, to prevent peritoneal adhesion formation, possible chronic pelvic pain, and infertility. In the present case, an active bleeding secondary to a perforation of the uterus during curettage, for diagnosis of endometrial carcinoma, was instantaneously and successfully treated with only the application of a novel modified polysaccharide powder. This is, to the best of our knowledge, the first time that the agent 4DryField® has been used for this purpose.

**Case presentation:**

A 71-year-old German woman with serometra and endometrial hyperplasia suffered a perforation of the anterior wall of the uterus during the hysteroscopic resection of submucosal polyps and a fractional curettage. Subsequently, an immediate laparoscopy showed an active bleeding from the wound, which was promptly stopped with only the application of the hemostatic and anti-adhesion polysaccharide powder, 4DryField®. There were no postoperative complications. Nine weeks later, a laparoscopic hysterectomy with bilateral salpingoophorectomy for endometrial carcinoma (histology: stage IA, pT1a, cN0, L0 V0 M0/G2) was performed. The former injured area looked slightly prominent, was completely healed, and showed a shiny serosa. All her pelvic organs were free of adhesions, and there was one 0.5-mm calcified granuloma in the Douglas pouch.

**Conclusions:**

The efficient hemostasis combined with the adhesion prevention effect of 4DryField®, allowed a fast control of the uterine wall bleeding, saved operation time, avoided the risks of other procedures for bleeding control and contributed to the normal healing of the uterine wall without any adhesion formation.

## Background

Hysteroscopy is a useful and routine diagnostic and therapeutic procedure, but some complications related with intrinsic factors of the patient and the procedure exist. Recognized risk factors for those undesirable events are: age of the patient, uterine conditions, small uterus, stenotic cervix, prior use of gonadotropin release hormone (GNRH) analogs, use of anesthesia or analgesia, patient positioning, extent of the surgery, type of the distending medium, use of thermal energy sources, poor visualization, and lack of uterine distension [1, 2]. Derived complications include perforation of the uterus, intrauterine bleeding, infection, burns, or air embolism and fluid overload syndrome. The latter two depend on the pressure, type, and amount of the distending medium used during the procedure [2]. If additional interventions are necessary to repair organs injured during hysteroscopy, other complications can occur related to the repairing surgeries. In addition, sometimes these complications are recognized late, or are life-threating for the patient [1–3].

The most common of the complications mentioned is uterine perforation, resulting in an inability to maintain a distended uterus. This event is reported to occur in between 0.12 and 1.4 % of hysteroscopies in German and American studies, respectively [4]. The lesion can occur during the dilatation of the cervix, curettage of the endometrium, or during the resection of septum, polyps, or leiomyomata. Small perforations usually are not life-threatening and can be treated with antibiotics and overnight observation. Greater lesions can produce acute bleeding of the injured area, leading to hemoperitoneum, rupture of uterine vessels causing hypovolemic shock or damage to adjacent bowel, with risk of peritonitis [1–3]. Therefore, following the German guidelines, a diagnostic laparoscopy rather than an echography is recommended in every perforation to evaluate the extent and control of the injury such as coagulation or suture of the uterine wall, hysterectomy, repair of the affected organs, or a combination of these procedures [5].

Consequently, the risk of postsurgical peritoneal adhesions, chronic pain, or infertility is increased. Specially, postsurgical peritoneal adhesions constitute a major problem in terms of patient quality of life and costs for the health system [6–8], with a high risk of intraoperative complications during a subsequent operation, if an adhesiolysis is performed. Hence it is recommended that surgeons adopt and implement anti-adhesion strategies, like the use of adhesion-reducing agents [9, 10].

## Case presentation

A 71-year-old German woman presenting with serometra and endometrial hyperplasia was scheduled to undergo diagnostic hysteroscopy and fractional curettage. She had delivered twice, and had a history of multiple previous surgeries including appendectomy, cholecystectomy, diaphragm hernia repair, and two former curettages because of postmenopausal abnormal bleeding. At physical examination, her genital organs were atrophic and no masses were palpable. The ultrasound showed a 6-cm-long uterus with a 1 cm hyperechogenic endometrium and serometra. Our patient was scheduled for a hysteroscopy under general anesthesia.

**Fig. 1:**
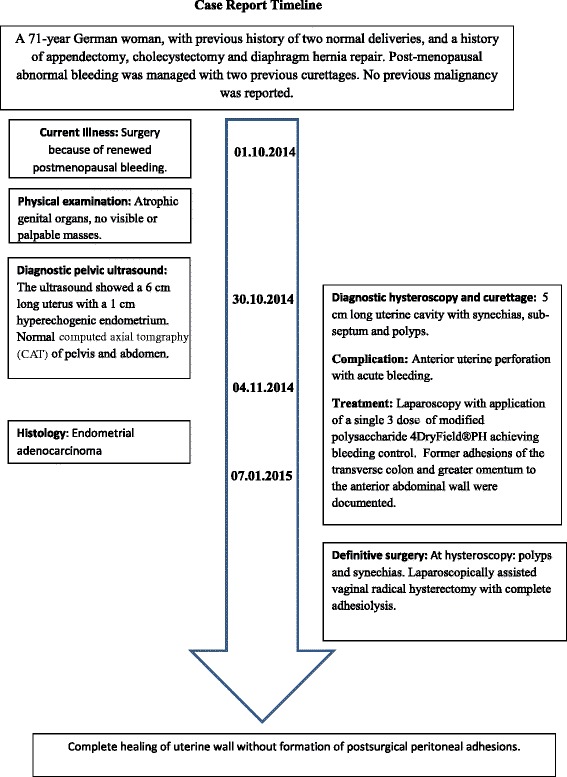
Timeline of interventions and outcomes

Because of a stenotic cervix, a dilatation to 0.7 cm was performed. The inspection revealed a 5-cm-long intrauterine cavity, with a subseptum, synechia in the fundus area, and submucosal polyps, which were resected. During the final fractional curettage the anterior wall of the uterus was perforated with the 0.7 cm curette, and an urgent laparoscopy was carried out. The laparoscopic inspection revealed massive adhesions of the transverse colon and greater omentum to the anterior abdominal wall, including formation of several adhesion bands, which were related to the previous abdominal surgeries. The bladder, bowel, and uterine vessels were intact, and the uterine perforation was confirmed. The active bleeding from the 1 cm wound was controlled in a few minutes by a single dose of 4DryField® powder. Consequently, further actions were not necessary and the postoperative patient recovery was satisfactory (Fig. 1).

**Fig. 2:**
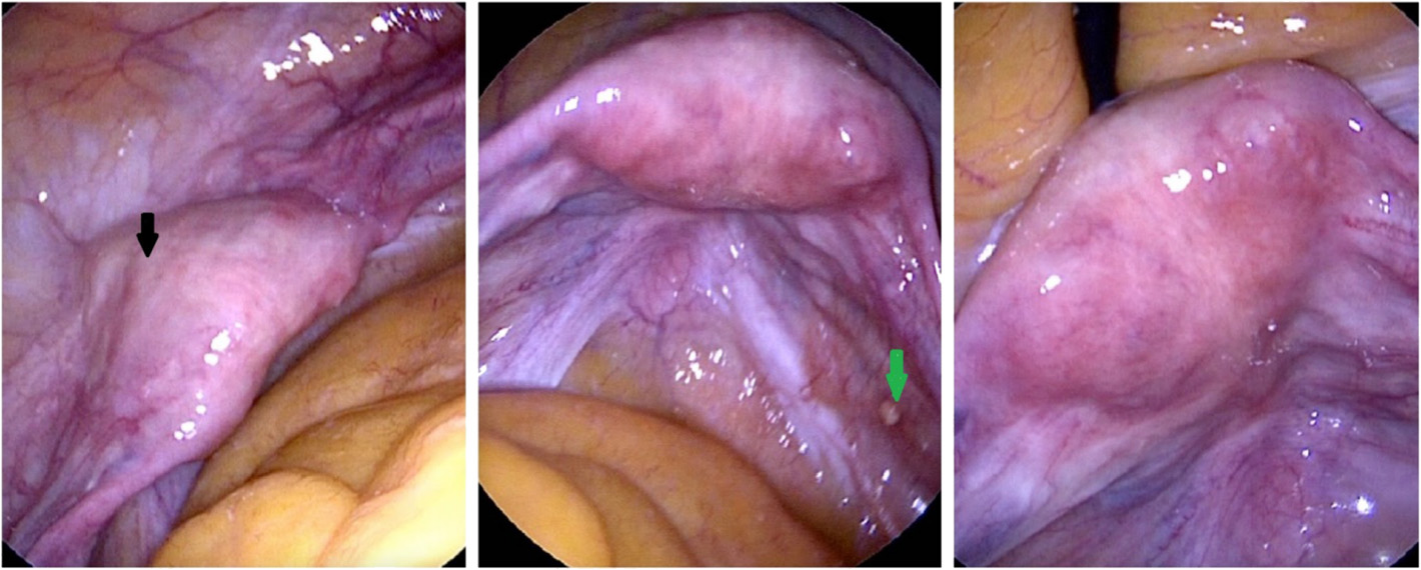
Aspect of the uterus 9 weeks after application of the modified polysaccharide 4DryField®. The area of the former uterine perforation is completely healed without adhesion formation, but is more prominent (*black arrow*). There is a 0.5 mm white granuloma in the depth of the Douglas pouch, which was histologically reported as a foreign body reaction with regressive calcification (*green arrow*)

Nine weeks after curettage, a control hysteroscopy, adhesiolysis of the preexisting abdominal adhesions, and a laparoscopically assisted vaginal hysterectomy with bilateral salpingoophorectomy, because of an endometrial adenocarcinoma, were performed following the German guidelines. The uterine cavity exhibited multiple endometrial polyps, few synechias, and a completely healed wall. A total of 1.5 mL of ascites was found in the Douglas pouch. The pelvic organs were free of adhesions, and the uterine wall completely uneventful; the area of former perforation was prominent with the shiny surface of normal peritoneum. Despite the region having sustained a bleeding injury, there was no adhesion formation (Fig. 2). Additionally, a 0.5 cm white granuloma in the right Douglas pouch was excised. There were no intra or postoperative complications. The histology reported ascites cells without atypia, an endometrioid adenocarcinoma of the corpus uteri (staging IA, pT1a, cN0, L0 V0 M0/G2), and a granuloma with a foreign body reaction and regressive calcification. The tumor immunohistology was positive for estrogen (80 %) and progestogen (80 %) receptors. Our patient received no adjuvant therapy.

## Discussion

In the present case, a perforation of the uterus occurred after excision of polyps and fractional curettage, requiring a laparoscopy for hemostasis, but instead of coagulating or suturing the uterine wall, the bleeding wound was treated with the novel modified polysaccharide 4DryField® (PlantTec Medical GmbH, Bad Bevensen, Germany), which exhibits a hemostatic and anti-adhesion double effect [11–15]. When this powder is directly applied onto the bleeding surface, it builds a tight viscous mesh of gel and blood components comparable to that of a native coagulum, and is capable of sealing bleeding areas. Moreover, when a 9 % saline solution is added to the powder, it transforms into a viscous gel, acting as a barrier for adhesion prevention. Since the postoperative diagnosis of endometrial carcinoma necessitated subsequent surgery, the outcome of this polysaccharide could be evaluated 9 weeks later. This is, to the best of our knowledge, the first report on the use of this substance to control the bleeding after a uterine perforation.

Perforation of the uterus is the most common complication of curettage and may result in several problems, including bleeding, damage to viscera, and peritonitis [1–3], requiring a fast damage control. Furthermore, hematoma formation and any kind of peritoneal trauma due to coagulation or suture of the uterine wall might result in adhesion formation with pathologic sequelae, such as chronic pain, secondary infertility, or acute ileus [8, 9, 15].

In the present case, perforation of the uterus had resulted in persisting oozing of blood, as documented by laparoscopy, which instantaneously and successfully could be treated with an application of an hemostatic powder, the modified polysaccharide 4DryField®PH, avoiding coagulation or suture of the uterine wall. Nine weeks after the treatment, a subsequent hysteroscopy and a radical hysterectomy by laparoscopy was performed because of an endometrial carcinoma, allowing the assessment of the outcome of the product. It was observed that the wound had healed satisfactorily with a normal appearance of the uterine wall and serosa, without adhesions around the uterus or pelvic organs. In this case, the prompt control of the bleeding through an efficient hemostasis, combined with the adhesion prevention effect of 4DryField® [15] could contribute to the healing of the myometrium, also avoiding peritoneal adhesions in a short period of time. This single case cannot be a formal proof of the efficacy of the mentioned novel hemostatic and anti-adhesion agent, but may contribute to its relevant evidence.

## Conclusions

This is the very first time the modified polysaccharide powder 4DryField® was used in a case of uterine perforation, under the rationale of assuring a fast control of the bleeding, saving operation time, diminishing the risk of postsurgical adhesions, and avoiding the risks of other procedures for bleeding control of the uterine wall. Accordingly, based on the dual action of this powder, combining a hemostatic and adhesion prevention effect, this novel product can be considered in the treatment of limited injuries of the uterine wall. Of course, further studies are necessary to establish the superiority of this product over other alternatives in the treatment of limited injuries of the uterus.
